# Evaluation of a Product Derived from Porcine Plasma Protein and a Yeast Product with Similar Biological Activity in Diets of Growing Broilers

**DOI:** 10.3390/ani11061751

**Published:** 2021-06-11

**Authors:** Park Waldroup, Mathew Vaughn, James McNaughton

**Affiliations:** 1 Poultry Science Department, University of Arkansas, Fayetteville, AR 72701, USA; waldroup@uark.edu; 2 Puretein Bioscience, Minneapolis, MN 55416, USA; 3 AHPharma, Hebron, MD 21830, USA; mcnaughton@ahpharma.com

**Keywords:** broiler, growth, protein, carcass

## Abstract

**Simple Summary:**

A 42-day feeding trial was carried out in a commercial research institution to determine the efficacy of two bioactive protein products, betaGRO^®^ (BG) and celluTEIN^®^ (CT). The birds were grown under two different environments. One was a low stress environment using clean wood shavings, while the other was a high stress environment where used wood shavings were obtained from a commercial farm that had suffered high mortality to simulate a commercial environment. Growing birds in the high stress environment had a negative impact upon overall bird performance; however, the addition of either BG or CT successfully mitigated these detriments. Improvements in 42-day body weight and feed conversion were observed in chicks grown in both environments in response to the addition of either BG or CT. Birds fed BG and CT were more uniform than birds grown on control diets. Mortality of birds in the high stress environment was significantly reduced by the addition of BG or CT. Addition of BG and CT resulted in improvements in total carcass yield and breast meat yield. This was observed under both environmental conditions, and in a similar magnitude for both products.

**Abstract:**

The post-hatch period of a broiler is an integral point in their development and for the programming of musculoskeletal and immune cells. Therefore, the efficacy of two bioactive protein products, betaGRO^®^ (BG) and celluTEIN^®^ (CT) to impact post-hatch and lifelong development was evaluated. Birds were grown under a low stress environment using clean wood shavings litter and a high stress environment with used litter from a commercial farm that had suffered high mortality. Each additive was fed at 300 g/ton from day 1–14 and 50 g/ton from day 15–42. Growing birds in the high stress environment had a negative impact on performance (*p* < 0.05); however, addition of either BG or CT successfully mitigated the detriments of the high stress environment (*p* < 0.05), and in many cases, the performance was equal to or better than the performance of birds on the control diet in the low stress environment. Birds fed BG and CT experienced improvements in 42-day feed conversion, and were more uniform than birds grown consuming the control diet (*p* < 0.05). Mortality of birds in the high stress environment was reduced by addition of BG or CT (*p* < 0.05). Addition of BG and CT resulted in improvements in carcass and breast meat yield (*p* < 0.05). Together, these data suggest that BG and CT can be used interchangeably to improve broiler health and performance.

## 1. Introduction

The period after broilers hatch is critical for development and adaptation of the small intestine to nutritional changes [1,2,3,4,5]. Additionally, during the post-hatch period the broilers immune system is naïve and skeletal muscle has the greatest metabolic demand of any system. Post-hatch nutrition has the potential to program life-long immune and muscle metabolism. Restriction of energy and amino acid density during the first 14 days hinders production performance and exacerbates muscle myopathies [6,7,8], thus, implying the importance of the first 14 days of life on skeletal muscle development. Additionally, post-hatch feed restrictions result in the depression of myogenic signaling regulatory pathways [9,10]. For many years, spray-dried plasma (SDP) proteins have been widely used in diets for nursery pigs [11,12,13,14,15]. The SDP contains functional proteins including immunoglobulins and biologically active peptides that may play crucial roles in cellular signaling [16,17]. Recently, studies have evaluated the use of SDP in poultry diets with positive results [16,18,19,20]. These positive results include greater body weight gain, with a pronounced benefit during exposure to pathogenic bacteria [21,22,23]. The product betaGRO^®^ (BG) is porcine plasma that is processed to enhance the bioactivity of key metabolic proteins and celluTEIN^®^ (CT) is a proprietary yeast-based product that is blended with key amino acids to simulate the biological value of protein products. Recent reports show that BG stimulates skeletal muscle growth and immune cell metabolism in vitro [24,25]. Therefore, the objective of this study was to evaluate broiler growth under pathogenic stress in response to BG and CT.

## 2. Materials and Methods

### 2.1. Ethical Standards

All experimental procedures comply with the Guide for the Care and Use of Agricultural Animals in Research and Teaching (FASS, 2010) and were previously approved by the Institutional Animal Care and Use Committee (IACUC) of AHPharma (Hebron, MD, USA), Inc. for the safe and humane treatment of animals. Where historical data is available, the total number of birds employed were selected to achieve adequate statistical power (*p* > 0.80) while minimizing the number of animals subject to testing. Euthanasia was performed under the American Veterinary Medical Association (AVMA) Guidelines for the Euthanasia of Animals by trained personnel only [26,27].

### 2.2. Animals and Experimental Design

The study was conducted to evaluate the performance of broiler chickens fed diets with two different sources of bioactive proteins. These include BG, produced from porcine plasma by a proprietary process, and CT, a yeast-based product that contains critical bioactive proteins. The trial was carried out in a well-recognized commercial testing laboratory in the United States (AHPharma) to simulate a commercial environment. 

The products were evaluated under two scenarios where the only difference in the environment was the pathogen load in the litter: a low stress environment using clean wood shavings as litter, and a high stress environment, in which litter was obtained from a commercial farm that had previously suffered 5.5% mortality. In addition, 5 million coccidia oocysts (50% *Eimeria acervulina* and 50% *Eimeria maxima*) per pen were scattered in the litter of these pens, each pen was then walked with a clean pair of plastic boots to spread the litter equally. Pens were equipped with bedding dividers to ensure that the bedding and pathogens applied to each pen were contained within the pen where they were applied. Within each environmental scenario, one of 3 dietary treatments were applied; CON, a common basal diet (Table 1); BG, added to the basal diet at the rate of 300 g/ton in starter (1–14 d), 50 g/ton in grower (15–28 d) and finisher (29–42 d) diets; and CT, added to the basal diet at the rate of 300 g/ton in starter (1–14 d), 50 g/ton in grower (15–28 d) and finisher (29–42 d) diets. Nutritionally complete vitamin and trace mineral mixes were used to fortify the diets. 

Vitamin-Trace Mineral premix contained calcium carbonate, ferrous sulfate, manganese oxide, zinc oxide, copper sulfate, ethylenediamine dihydroiodide, sodium selenite, rice hulls, calcium carbonate, vitamin E supplement, niacin supplement, d-calcium pantothenate, riboflavin supplement, vitamin A supplement, vitamin D3 supplement, 25-hydroxyvitamin D3, mineral oil, pyridoxine hydrochloride, menadione nicotinamide bisulfite, thiamine mononitrate, biotin, vitamin B12 supplement, folic acid. 

The test period began on trial day 0 (day of hatch of chicks) and ended on trial day 42. The pen served as the experimental unit and each pen contained 52 mixed-sex Cobb broilers (50:50 ratio) randomly assigned into 12 replicates per group at a density of 0.77 m^2^ of floor space per broiler. Sixty-six chicks were placed in each pen on day 0 and the number reduced to 52 on day 1 after crop-fill measurements for individual birds and verifying gender by feather sexing. Ensuring that the chicks had eaten based on crop fill decreases the variation among treatments, allowing more precise evaluation of the feed additives being tested. Birds were not replaced for the remainder of the study. The chicks were observed daily for signs of unusual grow-out patterns or health problems. All birds received Coccidiosis vaccine in the hatchery. No antibiotics were administered during the entire trial. 

### 2.3. Growth Performance

Birds were individually weighed at 1, 7, 14, 28 and 42 d of age and feed consumption was determined. Feed consumption was measured on trial days 1–7, 1–14, 1–28 and 1–42, and was used to calculate the feed to gain ratio. Within each pen the coefficient of variation among weights was determined as an indication of broiler uniformity at 7, 14, 21, 28 and 42 d of age. The European Production Efficiency Factor (EPEF) is a formula that considers body weight gain, feed conversion, and mortality, resulting in a single value that gives an overall picture of performance [28].

EPEF = (average gain grams/day × % survival rate)/(feed conversion × 10)(1)

### 2.4. Carcass Characteristics

On day 42 of the trial, 5 males and 5 females per pen were humanely euthanized by electrical stunning and exsanguination to evaluate processing yields. The carcass, pectoralis major, pectoralis minor, whole breast, thigh, wing, leg, and abdominal fat weights were collected. Subsequently, the carcass yield, and a percentage of each of the measured parts was calculated as a percentage of the live weight. 

### 2.5. Intestinal Health

Salmonella incidence was tested (two males and two females per pen at 14 d and five males and five females per pen at 42 d to simulate counts required by USDA/FSIS at processing. Lesion scores were determined by the method of Johnson and Reid (1970) and numbers of Clostridium perfringes, *E. coli*, and aerobic plate count (APC) were determined. The prevalence of *Clostridium perfringes*, *E. coli*, and aerobic plate counts were transformed by log^10^ to make the values relevant for the statistical model used for analysis [28].

Also, at 14 d two intestinal samples were taken from two males and two females per pen. Samples were taken from two gut areas per bird, one at the distal end of the duodenal loop and the second from the ileum, approximately two inches anterior to Meckel’s diverticulum. Immediately after removal, tissue samples were placed in 10 times their volume of 10% buffered formalin and stored at room temperature to fix for 1 week. Samples were then reduced to a 1 cm luminal cross section and placed in a cassette for paraffin embedding. Samples were subsequently mounted on a slide and subjected to hematoxylin and eosin staining. Photomicrographs were captured using standard light microscopy (AmScope MR095, Irvine, CA, USA). The microscope and imaging software were calibrated with a certified calibration slide prior to analysis. Average villi height and crypt depth were measured in 10 well-formed villi per cross section.

### 2.6. Statistical Analysis

Statistical analyses were performed using the GLIMMIX Procedures of SAS 9.4 (Cary, NC, USA). Data were analyzed as a 2 × 3 factorial arrangement. The fixed effects were diet (DIET), environment (ENV) and the DIET × ENV interactions. Since day 1 body weights were different among treatments, the day 1 body weights served as a covariate for analysis for the rest of the parameters evaluated to ensure that any differences in starting weight did not influence response criteria. Pair-wise comparisons between the least square means of the factor levels were computed using the PDIFF option of the LSMEANS statement. Differences were considered significant at α ≤ 0.05.

## 3. Results

### 3.1. Growth Performance

Performance parameters are presented in Table 2. There was not an ENV × DIET interaction for body weight at day 7, 14 or 28 (*p* > 0.05); however, there was an interaction at trial day 42 where the magnitude of body weight increase in response to BG and CT was greater in the high stress environment compared to the respective controls in the low stress environment (*p* < 0.05). At trial day 7, ENV did not influence body weight (*p* > 0.05). At trial day 14, 28 and 42, broilers housed in the low stress environment had greater body weights compared to broilers in the high stress environment (*p* < 0.05). Broilers supplemented with BG and CT had greater body weights over the course of the trial compared to broilers fed the control diet (*p* < 0.05) but were similar to each other (*p* > 0.05). 

There was not an ENV × DIET interaction for feed consumption (*p* > 0.05). The ENV did not influence feed consumption between days 1–7 or 1–42 (*p* > 0.05); however, feed consumption was greater for broilers in the low stress environment compared to broilers in the high stress environment (*p* < 0.05). Feed consumption was greater during the trial intervals of 1–7, 1–14 and 1–28 days for broilers fed BG and CT compared to CON broilers (*p* < 0.05), with BG and CT broilers consuming similar amount of feed (*p* > 0.05). The DIET did not influence feed consumption during the trial interval of 1–42 days (*p* > 0.05).

There was not an ENV × DIET interaction for feed conversion of broilers over the course of the trial (*p* ≥ 0.07). Environment did not influence (*p* = 0.66) feed conversion between days 0 and 7; however, feed conversion in the low stress environment was improved between days 1–14, 1–28 and 1–42 compared to broilers raised in the high stress environment (*p* ≤ 0.01). Broilers supplemented with BG and CT had improved feed conversion compared to CON broilers at all time points (*p* < 0.05) but were similar to each other (*p* > 0.05).

There was not an ENV × DIET interaction on mortality during the intervals between days 1–7, 1–14 or 1–28 (*p* > 0.05). There was an ENV × DIET interaction during the interval of days 0–42 where dietary treatments had similar mortality in the low stress environment (*p* > 0.05), but mortality was improved in response to BG and CT supplementation in the high stress environment compared to broilers on the CON diet (*p <* 0.05). Mortality from days 1–7 was not influenced by ENV or DIET (*p* > 0.05). During the intervals 1–14, 1–28 and 1–42 days of the trial, broilers from the high stress environment experienced greater mortality than broilers raised in the low stress environment (*p* < 0.05). Additionally, broilers supplemented with BG and CT had a lower percentage of mortality during trial intervals 1–14, 1–28 and 1–42 days compared to broilers fed the control diet (*p* < 0.05), while broilers fed BG and CT had a similar level of mortality (*p* > 0.05).

Body weight uniformity is expressed as a percentage of the coefficient of variation of the body weight of broilers within treatment groups. At trial day 7 and 14 there was not an ENV × DIET interaction for body weight uniformity (*p* > 0.05). At both day 28 and 42 there was an ENV × DIET interaction where the magnitude of improvement in uniformity of BG and CT supplemented broilers compared to CON fed broilers was greater under the high stress environment compared to the low stress environment (*p* < 0.05). The ENV did not influence body weight variation at day 7 (*p >* 0.05). At days 14, 28 and 42, broilers grown in the low stress environment were more uniform compared to broilers grown in the high stress environment (*p* < 0.05). At all time-points broilers that were supplemented with BG and CT had a more uniform body weight compared to broilers fed the CON diet (*p* < 0.05), and broilers supplemented with BG and CT exhibited similar body weight uniformity (*p* > 0.05).

The EPEF is a formula that considers body weight gain, feed conversion, and mortality that results in a single value that gives an overall picture of performance. There was not a ENV × DIET interaction for the EPEF during the trial intervals of 1–7, 1–14 or 1–28 days (*p* > 0.05). Over the interval from 1–42 days there was an ENV × DIET interaction where the magnitude of improvement in response to BG and CT supplementation was greater in the high stress environment compared to the low stress environment (*p* < 0.05). The ENV did not influence the EPEF during the trial interval from day 1–7 (*p* > 0.05). During the remaining trial intervals, broilers grown in the low stress environment had a greater EPEF compared to the broilers grown in the high stress environment (*p* < 0.05). Over all intervals in the trial, broilers supplemented with BG and CT had a greater EPEF compared to CON fed broilers (*p* < 0.05), with BG and CT supplemented broilers having a similar EPEF (*p* > 0.05).

### 3.2. Carcass Characteristics

Carcass processing yields are presented in Table 3. There was not an interaction of ENV × DIET for the percentage of the carcass represented by the whole breast, thigh, wing, leg, or abdominal fat (*p* > 0.05). There was a ENV × DIET interaction for carcass yield, pectoralis major percentage, pectoralis major weight, pectoralis minor percentage, pectoralis minor weight, and whole breast weight, where meat yield increases in response to BG and CT supplementation were greater in magnitude for broilers raised in the high stress environment compared to those raised in the low stress environment (*p* < 0.05). The ENV did not influence the percentage of the carcass represented by the thigh, wing, leg, or abdominal fat (*p* > 0.05). Broilers grown in the low stress environment had a greater carcass yield, pectoralis major percentage, pectoralis major weight, pectoralis minor percentage, pectoralis minor weight, whole breast percentage, and whole breast weight compared to broilers grown in the high stress environment (*p* < 0.05). There was no difference in carcass proportions between groups of broilers supplemented with BG or CT (*p* > 0.05). Broilers that were supplemented with BG and CT had an increased chilled carcass yield, pectoralis major percentage, pectoralis major weight, pectoralis minor percentage, pectoralis minor weight, whole breast percentage, and whole breast weight compared to CON broilers (*p* < 0.05). The DIET did not affect the percentage of carcass represented by the thigh, wing, leg, or abdominal fat (*p* > 0.05). 

### 3.3. Intestinal Health

Gut morphology metrics evaluated at day 14 and 42 of the study are presented in Table 4; there was not an ENV × DIET interaction for villi height, ratio of villi height to crypt depth, log formations of APC, or oocysts present in the feces (*p* > 0.05). There was a similar ENV × DIET interaction for lesion score, crypt depth, *E. coli* presence, *Clostridium perfringens*, and salmonella where the BG, CT, and CON broilers were similar in the low stress environment, but BG and CT supplementation improved these gut health measures in the high stress environment compared to CON broilers (*p* < 0.05). The main effect of ENV did not influence the crypt depth, villi height to crypt depth ratio, APC, or oocysts presence in the feces (*p* > 0.05). Broilers in the high stress environment had greater lesion scores, *E. coli* prevalence, *Clostridium perfringens* prevalence, percentage of salmonella, and decreased villi height compared to the broilers grown in the low stress environment (*p* < 0.05). DIET did not influence the villi height, crypt depth, villi height to crypt depth ratio, *E. coli* prevalence, APC prevalence, salmonella incidence, or oocysts in the feces (*p* > 0.05). Additionally, gut morphology and bacterial presence were similar for all parameters evaluated among BG and CT supplemented broilers (*p* > 0.05); however, BG and CT supplemented broilers had decreased lesion scores, and decreased *Clostridium perfringens* compared to CON fed broilers (*p* < 0.05). 

## 4. Discussion

Numerous authors have observed improvements in body weight and feed conversion when fed diets containing porcine or bovine plasma protein [16,18,19,20]. Spray dried plasma proteins are a good source of protein and contain specific bioactive proteins that aid in animal growth [16,18,19,20]. In the current study, birds also experienced improvements in body weight and feed conversion in response to BG and CT supplementation, which is likely due to many of the bioactive proteins contained in these products (17). Additionally, almost every production measurement was negatively impacted by the high stress environment. Similar to plasma supplementation, the magnitude of the performance improvements was greater in response to BG and CT under challenged conditions [20]. In the low stress environment, at 42 d, birds fed BG and CT were 192 g and 175 g heavier than the low stress control, respectively. In the high stress environment, at 42 d, birds fed BG and CT were 260 g and 262 g heavier than the high stress control, respectively. The improved gains in BG and CT fed birds also resulted in similar results for the feed conversion ratio. 

In the high stress environment, birds fed BG and CT had lower lesion scores compared to the high stress control. These improvements observed in the gut morphology are in agreement with previous results [21,22]. The improvement in performance of birds fed BG and CT during exposure to highly pathogenic bacteria mimics the response previously observed in response to plasma supplementation [23]. Birds fed under the high stress environment had greater numbers of *E. coli*, salmonella, and clostridia, but these were reduced in birds fed BG and CT, which likely contributed to the improved gut morphology observed in the current study. 

Many of the responses observed in the current research to the supplementation of BG and CT are consistent with data previously reported when broilers were supplemented with SDP [16,18,19,20,21,22,23]; however, the inclusion rate of BG and CT into the diets was only 1.5–6 percent of the inclusion rate of SDP [21]. Therefore, the response to BG and CT supplementation is likely not nutritive but due to metabolic improvements. Recent research has demonstrated that BG promoted immune cell function and skeletal muscle growth in vitro [24,25]. Inclusion of BG in culture medium at 10 mg/mL resulted in significantly larger myotube size and was mediated by positive changes in mechanistic target of rapamycin (mTOR) signaling proteins [24]. Immune research previously conducted observed that BG supplementation increased respiratory metabolism of actively growing β-lymphocyte cells by more than two-fold, which was mediated by mTOR pathway signaling since the addition of rapamycin abolished all positive treatment effects [25]. This provides a likely mode of action for many of the bioactive components that are contained in plasma, BG, and CT. This, in conjunction with the current data, suggests that supplementation of BG and CT in a high stress environment bolsters the broilers’ immune system to mitigate bacterial interference in the small intestine, which results in performance detriments, while simultaneously promoting superior lean tissue growth. 

## 5. Conclusions

The addition of BG and CT improved growth rate, feed conversion, livability, dressing percentage, and breast meat yield of broilers. Almost all production parameters were negatively impacted by a stressful environment. It is noteworthy that BG and CT supplementation was able to mitigate the performance detriments associated with the high stress environment. Much of the improved performance observed in broilers in this study is likely due to improvements in cellular metabolism and programing. Supplementation with these bioactive peptides from both commercial sources appears to stimulate the immune response needed to overcome the pathogen challenge presented in this study. Additionally, skeletal muscle growth was increased when broilers were fed BG and CT. The performance improvements observed were similar when birds were fed BG and CT. These data suggest that BG and CT have potential to improve immunity and production performance in the commercial broiler industry.

## Figures and Tables

**Table 1:** Feed composition (%) and formulated nutrient profile of diets.

Ingredient	Starter	Grower	Finisher
	0–14 d	14–28 d	28–42 d
Yellow corn	45.7	51.4	55.4
Soybean meal 47%	38.3	34.5	30.7
Soybean oil	8.69	8.04	8.26
Blended animal protein ^1^	2.00	2.00	2.00
DL Methionine	0.439	0.260	0.190
Salt	0.599	0.549	0.499
L-Lysine HCl	0.088	0.034	0.042
Limestone	1.46	1.27	1.20
Dicalcium phosphate	2.33	1.60	1.47
Choline CL 60%	0.112	0.067	0.026
Vitamin-Trace minerals ^2^	0.100	0.100	0.100
Phytase	0.100	0.100	0.100
Crude protein%	23.0	21.5	20.0
ME kcal/lb	1425	1450	1475
Lysine%	1.40	1.25	1.15
TSAA% ^3^	1.15	0.950	0.850
Methionine%	0.788	0.599	0.513
Calcium%	1.10	0.900	0.840
Available P%	0.600	0.450	0.420
Total P%	0.904	0.734	0.692
Sodium%	0.260	0.240	0.220

^1^ Nutrient values are calculated and presented on an as fed basis. ^2^ Vitamin-Trace Mineral premix contained calcium carbonate, ferrous sulfate, manganese oxide, zinc oxide, copper sulfate, ethylenediamine dihydroiodide, sodium selenite, rice hulls, calcium carbonate, vitamin E supplement, niacin supplement, d-calcium pantothenate, riboflavin supplement, vitamin A supplement, vitamin D3 supplement, 25-hydroxyvitamin D3, mineral oil, pyridoxine hydrochloride, menadione nicotinamide bisulfite, thiamine mononitrate, biotin, vitamin B12 supplement, folic acid. CON = complete diet without either feed supplement; BG = base feed supplemented with 300 ppm betaGRO on days 0–14 and 50 ppm on days 15–42; CT = base feed supplemented with 300 ppm celluTEIN on days 0–14 and 50 ppm on days 15–42. ^3^ TSAA% is the percentage of total sulfur containing amino acids.

**Table 2:** Live performance of birds fed betaGRO and celluTEIN under different environmental conditions.

Measurement	Treatment ^1^						SEM	*p-*Value		
	Low Stress Environment			High Stress Environment						
	CON	BG	CT	CON	BG	CT		ENV × DIET	ENV	DIET
Average body weight (g)										
Day 1	56.0 ^b^	59.8 ^a^	59.8 ^a^	56.1 ^b^	60.2 ^a^	59.3 ^a^	0.26	0.227	0.988	<0.001
Day 7	168.3	189.9	190.2	169.7	191.0	191.1	1.19	0.947	0.082	<0.001
Day 14	451.0	525.7	529.7	414.9	492.0	486.9	4.85	0.366	<0.001	<0.001
Day 28	1276.6	1472.0	1470.4	1186.4	1372.6	1363.2	11.29	0.511	<0.001	<0.001
Day 42	2447.1 ^c^	2656.5 ^a^	2639.8 ^a^	2273.1 ^d^	2551.5 ^b^	2549.4 ^b^	19.76	0.004	<0.001	<0.001
Feed Consumption, (g)										
Days 1–7	14.90	16.27	16.39	14.74	16.44	16.71	0.26	0.355	0.408	<0.001
Days 1–14	31.50	35.43	35.45	29.98	33.17	32.83	0.62	0.380	<0.001	<0.001
Days 1–28	63.08	70.05	69.63	62.11	67.55	67.69	1.07	0.528	0.002	<0.001
Days 1–42	106.76	111.60	110.31	106.78	109.84	110.54	1.84	0.660	0.601	0.261
Average feed Conversion										
Days 1–7	0.94	0.87	0.87	0.92	0.87	0.88	0.011	0.127	0.663	0.001
Days 1–14	1.12	1.06	1.05	1.16	1.07	1.07	0.011	0.114	0.008	<0.001
Days 1–28	1.41	1.35	1.35	1.48	1.40	1.41	0.019	0.500	<0.001	0.015
Days 1–42	1.85	1.78	1.78	1.95	1.81	1.82	0.026	0.071	<0.001	0.005
Mortality, %										
Days 1–7	1.42	0.01	0.02	1.36	0.03	0.05	0.36	0.977	0.955	0.013
Days 1–14	1.93	0.14	0.17	4.76	1.61	2.12	0.65	0.259	<0.001	0.020
Days 1–28	1.86	0.63	0.81	6.69	3.51	3.68	0.79	0.090	<0.001	0.104
Days 1–42	2.10 ^a^	1.22 ^a^	0.87 ^a^	8.33 ^c^	4.69 ^b^	4.69 ^b^	0.92	0.014	<0.001	0.050
Uniformity, %CV										
Day 7	14.65	10.07	10.21	14.89	10.18	10.38	0.29	0.946	0.277	<0.001
Day 14	18.15	12.07	12.61	20.50	14.00	13.62	0.49	0.105	<0.001	<0.001
Day 28	15.81 ^c^	9.65 ^a^	9.69 ^a^	18.79 ^d^	11.03 ^b^	10.88 ^b^	0.50	0.013	<0.001	<0.001
Day 42	12.27 ^b^	10.54 ^a^	10.12 ^a^	17.18 ^c^	10.94 ^ab^	10.87 ^ab^	0.58	<0.001	<0.001	<0.001
European Production Efficiency Factor										
Days 1–7	249.61	312.93	311.94	257.61	314.19	309.45	4.93	0.256	0.384	<0.001
Days 1–14	280.77	353.18	358.68	237.70	322.29	316.73	5.38	0.160	<0.001	<0.001
Days 1–28	307.69	375.14	375.86	254.36	326.93	321.20	6.17	0.697	<0.001	<0.001
Days 1–42	304.09 ^b^	346.17 ^a^	347.67 ^a^	242.96 ^c^	313.02 ^b^	310.00 ^b^	6.08	0.001	<0.001	<0.001

ENV = Environmental effect; ^a–d^ Means within a row with different superscripts differ. ^1^ In the low stress environment broilers were housed in clean pens with new wood shavings; in the high stress environment broilers were housed in pens bedded with litter from a farm with high incidence of mortality and overseeded with Clostridium spores and coccidial oocysts; CON = complete diet without either feed supplement; BG = base feed supplemented with 300 ppm betaGRO on days 0–14 and 50 ppm on days 15–42; CT = base feed supplemented with 300 ppm celluTEIN on days 0–14 and 50 ppm on days 15–42.

**Table 3:** Forty-two-day processing yields of birds fed betaGRO and celluTEIN under different environmental conditions.

Measurement	Treatment ^1^						SEM	*p-*Value		
	Low Stress Environment			High Stress Environment						
	CON	BG	CT	CON	BG	CT		ENV × DIET	ENV	DIET
Carcass Yield (% post chill)	75.63 ^a^	76.50 ^a^	75.53 ^a^	72.74 ^b^	76.18 ^a^	76.30 ^a^	0.61	<0.001	0.013	0.038
Pectoralis Major (% post chill)	19.83 ^c^	20.60 ^b^	20.92 ^a^	18.59 ^d^	20.34 ^bc^	20.34 ^b^	0.18	<0.001	<0.001	<0.001
Pectoralis Major, g	385.33 ^c^	436.67 ^a^	436.27 ^a^	323.78 ^d^	411.42 ^b^	413.84 ^a^	4.73	<0.001	<0.001	<0.001
Pectoralis Minor (% post chill)	4.99 ^b^	5.76 ^a^	5.81 ^a^	4.68 ^c^	5.03 ^b^	5.13 ^b^	0.07	<0.001	<0.001	<0.001
Pectoralis Minor, g	96.91 ^c^	122.11 ^a^	121.11 ^a^	81.67 ^d^	101.78 ^b^	104.26 ^b^	1.49	0.030	<0.001	<0.001
Whole Breast (% post chill)	24.82	26.36	26.73	23.27	25.37	25.46	0.23	0.167	<0.001	<0.001
Whole Breast, g	482.24 ^c^	558.78 ^a^	557.38 ^a^	405.45 ^d^	513.21 ^b^	518.10 ^b^	5.91	<0.001	<0.001	<0.001
Thigh Yield (% post chill)	14.00	13.85	13.64	14.43	13.72	13.55	0.33	0.353	0.689	0.224
Wing Yield (% post chill)	10.38	9.67	10.12	10.57	9.60	9.72	0.29	0.289	0.530	0.091
Leg Yield (% post chill)	12.49	12.37	13.01	12.80	12.66	12.52	0.28	0.054	0.819	0.405
Abdominal Fat (% post chill)	1.31	1.33	1.34	1.37	1.32	1.30	0.03	0.090	0.833	0.913

ENV = Environmental effect; ^a–d^ Means within a row with different superscripts differ. ^1^ In the low stress environment broilers were housed in clean pens with new wood shavings; in the high stress environment broilers were housed in pens bedded with litter from a house with high incidence of mortality and overseeded with Clostridium spores and coccidial oocysts; CON = complete diet without either feed supplement; BG = base feed supplemented with 300 ppm betaGRO on days 0–14 and 50 ppm on days 15–42; CT = base feed supplemented with 300 ppm celluTEIN on days 0–14 and 50 ppm on days 15–42.

**Table 4:** Intestinal measurements of birds fed betaGRO and celluTEIN under different environmental conditions.

Measurement	Treatment ^1^						SEM	*p-*Value		
	Low Stress Environment			High Stress Environment						
	CON	BG	CT	CON	BG	CT		ENV × DIET	ENV	DIET
Lesion Score, 14 d	0.44 ^a^	0.08 ^a^	0.21 ^a^	1.85 ^c^	0.87 ^b^	0.87 ^b^	0.15	<0.001	<0.001	0.004
Lesion Score, 42 d	0.33 ^a^	0.24 ^a^	0.22 ^a^	1.64 ^c^	0.67 ^b^	0.59 ^b^	0.06	<0.001	<0.001	<0.001
Villi Height (µm) 14 d	1011.29	1022.89	1012.19	950.95	996.04	975.88	26.19	0.590	0.004	0.602
Crypt Depth (µm) 14 d	434.02 ^a^	468.45 ^a^	468.56 ^a^	377.01 ^b^	481.39 ^a^	450.35 ^a^	20.78	0.037	0.060	0.054
Villi Height: Crypt Depth Ratio	2.45	2.26	2.22	2.71	2.14	2.26	0.15	0.150	0.441	0.178
*E. coli* (log_10_) 14 d	4.54 ^a^	5.13 ^b^	5.29 ^b^	6.44 ^d^	6.32 ^cd^	6.13 ^c^	0.19	<0.001	<0.001	0.636
*E. coli* (log_10_) 42 d	4.79 ^a^	5.19 ^b^	5.18 ^b^	6.56 ^d^	6.26 ^cd^	6.08 ^c^	0.13	<0.001	<0.001	0.521
APC (log_10_) 14 d	8.23	8.06	8.10	8.47	7.84	7.96	0.37	0.601	0.851	0.728
APC (log_10_) 42 d	8.38	8.05	8.00	8.39	8.19	8.01	0.20	0.835	0.621	0.238
*Clostridium perfringens* (log_10_) 14 d	3.08 ^abc^	2.84 ^ab^	2.67 ^a^	4.54 ^d^	3.25 ^c^	3.10 ^bc^	0.21	<0.001	<0.001	0.001
*Clostridium perfringens* (log_10_) 42 d	2.95 ^a^	2.82 ^a^	2.76 ^a^	4.28 ^c^	3.30 ^b^	3.38 ^b^	0.11	<0.001	<0.001	<0.001
*Salmonella* Incidence, % 14 d	20.24 ^a^	25.30 ^a^	23.21 ^a^	80.70 ^c^	52.47 ^b^	62.67 ^b^	7.76	0.005	<0.001	0.514
*Salmonella* Incidence, % 42 d	9.68 ^a^	16.47 ^a^	13.01 ^a^	70.07 ^c^	48.17 ^b^	48.44 ^b^	5.74	<0.001	<0.001	0.408
Oocysts/g feces 14 d	6.24	5.97	6.07	6.36	5.72	5.98	0.21	0.399	0.515	0.237
Oocysts/g feces, 42 d	6.34	6.07	6.16	6.25	5.87	6.08	0.16	0.313	0.139	0.428

ENV = Environmental effect; ^a–d^ Means within a row with different superscripts differ. ^1^ In the low stress environment broilers were housed in clean pens with new wood shavings; in the high stress environment broilers were housed in pens bedded with litter from a house with high incidence of mortality and overseeded with Clostridium spores and coccidial oocysts; CON = complete diet without either feed supplement; BG = base feed supplemented with 300 ppm betaGRO on days 0–14 and 50 ppm on days 15–42; CT = base feed supplemented with 300 ppm celluTEIN on days 0–14 and 50 ppm on days 15–42.

## Data Availability

The data presented in this study are available on request from the corresponding author. The data are not publicly available due to the large quantity and complexity of the raw data.
